# Low anterior resection with transanal transection and single-stapled anastomosis: technical aspects and initial results

**DOI:** 10.1007/s00384-024-04646-3

**Published:** 2024-06-05

**Authors:** Alfredo Vivas López, Oscar Garcia Villar, Javier Garcia Borda, Rafael Restrepo Nuñez, Eduardo Rubio, Cristina Nevado, Pablo Pelaez, Maria Labalde Martinez, David Alias, Kleber Falcon, Sofia Lorenzo, José Perea García, Eduardo Ferrero

**Affiliations:** 1 Surgery Department, 12 de Octubre University Hospital, Madrid, Spain; 2 Surgery Department, Vithas Arturo Soria Hospital, Madrid, Spain; 3 Molecular Medicine Unit, Department of Medicine, Institute of Biomedical Research of Salamanca (IBSAL), Institute of Molecular and Cellular Biology of Cancer (IBMCC), Campus Miguel de Unamuno s/n, University of Salamanca-SACYL-CSIC, 37007 Salamanca, Spain

**Keywords:** Rectal cancer surgery, TTSS, Anastomotic leak, Single-stapled

## Abstract

**Background:**

Rectal cancer (RC) is a surgical challenge due to its technical complexity. The double-stapled (DS) technique, a standard for colorectal anastomosis, has been associated with notable drawbacks, including a high incidence of anastomotic leak (AL). Low anterior resection with transanal transection and single-stapled (TTSS) anastomosis has emerged to mitigate those drawbacks.

**Methods:**

Observational study in which it described the technical aspects and results of the initial group of patients with medium-low RC undergoing elective laparoscopic total mesorectal excision (TME) and TTSS.

**Results:**

Twenty-two patients were included in the series. Favourable postoperative outcomes with a median length of stay of 5 days and an AL incidence of 9.1%. Importantly, all patients achieved complete mesorectal excision with tumour-free margins, and no mortalities were reported.

**Conclusion:**

TTSS emerges as a promising alternative for patients with middle and lower rectal tumours, offering potential benefits in terms of morbidity reduction and oncological integrity compared with other techniques.

## Introduction

With an incidence in the European Union of approximately 125,000 cases per year (and increasing) [1], rectal cancer is a common pathology, but its surgical treatment can be very demanding and technically complex [2].

For tumours of the middle and lower rectum, treatment involves the removal of the rectum affected by the tumour and total excision of the mesorectum (TME). This procedure can be performed by open, laparoscopic, or robotic approaches with similar oncological results [3]. There are considerable differences in the oncological results and perioperative morbidity and mortality rates associated with the abdominal or transanal approach for rectal cancer resection and the preparation of the anastomosis [4].

In the case of anastomosis, the most standardized technique is double stapled (DS), in which the distal section is created with the help of a linear stapling device, and on that stapled line, a circular anastomosis is made with the help of a second stapled device. This approach appears to have multiple disadvantages that could increase the incidence of anastomotic complications, with published anastomotic leak (AL) rates ranging from 8 to 30% [5, 6]. For this reason, multiple options have been tested to decrease the incidence of AL, among which the techniques with only one stapled line are the most notable: total mesorectal excision via the transanal route (TaTME) and, more recently, low anterior resection via transanal transection with single stapled (TTSS), developed by Prof. A. Spinelli [7].

The TTSS technique allows dissection of the mesorectum via the abdominal route via an open, laparoscopic or robotic approach (without requiring additional training to achieve proficiency, unlike the TATME technique); rectal transection via the endorectal route, which allows control of the distal margin of the section; and subsequently, preparation of an anastomosis via single stapled and double stapled, with AL rates reported in the first series of 6.48% vs. 15.28%, respectively [8].

The aim of this publication is to describe the technical aspects and results in terms of oncological outcomes and perioperative morbidity and mortality rates in the first 22 patients who underwent surgery via the TTSS technique at our centre and to confirm the viability and reproducibility of the procedure.

## Methods

The study will be implemented and reported in line with the STROBE (STrengthening the Reporting of OBservational studies in Epidemiology) statement.

### Patients

A prospective observational study of the perioperative and oncological outcomes in 22 patients with medium or low rectal neoplasia who underwent low and ultralow anterior resection with TME and TTSS was conducted at two centres between December 2022 and October 2023. All surgeries were performed by the same team of two colorectal surgeons.

A diagnosis was made by flexible endoscopy, histological confirmation, thoraco-abdomino-pelvic tomography, pelvic magnetic resonance imaging for staging in all patients, and endoscopic ultrasound in the selected patients. Prior to surgery, all patients were evaluated by a multidisciplinary committee, in which surgical treatment and the option of adjuvant chemotherapy and radiotherapy (Rt) were decided upon where indicated (Rt + FOLFOX or CAPEOX treatment).

### Variables

The main variable in the study was the occurrence of AL, which was defined as leakage of luminal contents from a surgical junction between two hollow viscera [9] and was diagnosed by the presence of intestinal contents in the abdominal drainage tube and/or by confirmation via an imaging test.

In addition, demographic variables (sex, age, BMI, comorbidities) were recorded, as were variables related to tumour characteristics (distance from the margin), surgical procedure (approach and operative time), and postoperative evolution (mean length of stay, complications, readmission).

### Surgical technique

**Fig. 1:**
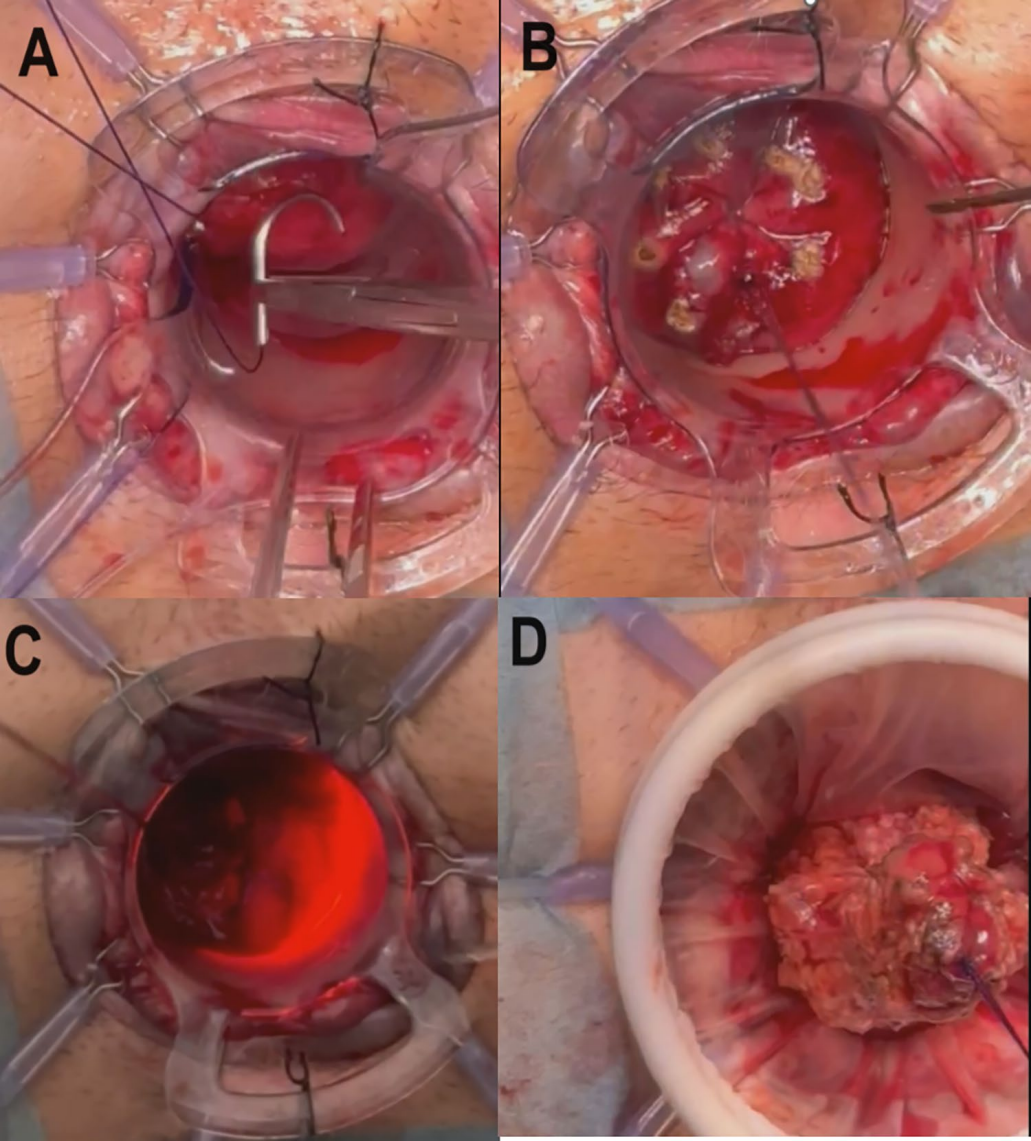
Steps of TTSS. **A** The first purse string (0/polypropylene) suture was placed distal to the tumour and transanally at a safe distance from the tumour. **B** The mucosa distal to the closure of the first tobacco pouch was marked. **C** Full-thickness circumferential rectotomy was performed using monopolar energy, facilitated by transillumination from the abdominal cavity. **D** Once the rectotomy was finished, a wound shield was placed before the sample was extracted, either transanally or transabdominally

**Fig. 2:**
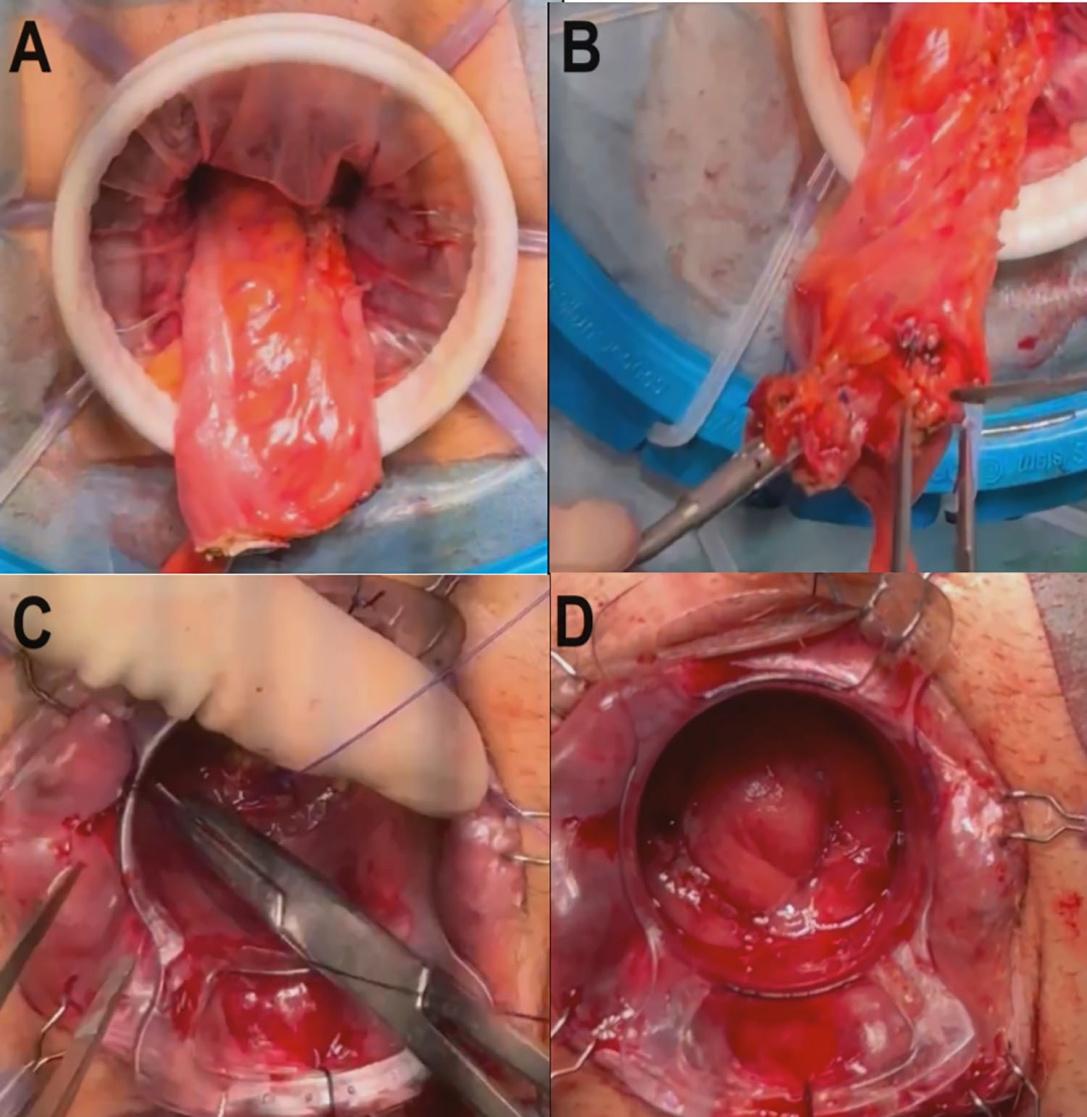
Steps of TTSS. **A** Completed section of the colon. **B** The circular anvil of the stapler (CSC-KOL^®^ 29 mm, B. Braun) was placed and secured in the proximal sectioned colon, and the colon was repositioned towards the pelvis. **C **The tractors of the Lone-Star^®^ were repositioned and placed at the level of the external margin. The 34-mm anoscope was repositioned again, and a second purse-string (0/polypropylene) suture was made, this time in the distal rectum. **D **The stapler was attached to the anvil, and a single-stapled anastomosis was created, with the anoscope still in place for optimal control

The laparoscopic lower anterior resection was performed following the usual technique in all cases [10], and the TTSS technique was performed following the steps published by Spinelli et al. [11], (before performing the first case, one of the surgeons on the team spent 4 weeks with Professor Spinelli at the Humanitas Hospital, in Milan, to learn the technique and steps) with some modifications made in our case (no need to use a drainage tube thanks to the long anvil of the CSC-KOL^®^ stapler):

1. Complete mesorectal dissection is completed via the abdominal approach (open, laparoscopic, or robot-assisted) until the upper third of the canal is reached when the lower margin of the tumour is exceeded during dissection.
2. A Lone-Star^®^ retractor (at the level of the pectineal line or slightly proximal) and a 34-mm cylindrical anoscope (model CK34M; Frankenman™) were placed in the canal, after which the lumen was washed with an iodinated solution.
3. The first purse string (0/polypropylene) suture was placed distal to the tumour and transanally at a safe distance from the tumour (Fig. 1a).
4. The purse string suture was used to close the rectum, taking care to ensure the wound was airtight to reduce the risk of tumour spread (Fig. 1a).
5. The mucosa distal to the closure of the first tobacco pouch was marked, and full-thickness circumferential rectotomy was performed using monopolar energy, facilitated by transillumination from the abdominal cavity (Fig. 1b).
6. Once the rectotomy was finished, a wound shield was placed before the sample was extracted, either transanally or transabdominally (Fig. 1c and d).
7. The circular anvil of the stapler (CSC-KOL^®^ 29 mm, B. Braun) was placed and secured in the proximal sectioned colon, and the colon was repositioned towards the pelvis (Fig. 2a and b).
8. The tractors of the Lone-Star^®^ were repositioned and placed at the level of the external margin. The 34 mm anoscope was repositioned again, and a second purse-string (0/polypropylene) suture was made, this time in the distal rectum (Fig. 2c).
9. The long anvil of the CSC-KOL stapler was pulled, completing the second purse-string suture and taking care to ensure that the stapler was also as airtight as possible.
10. The stapler was attached to the anvil, and a single-stapled anastomosis was created, with the anoscope still in place for optimal control (Fig. 2d).
11. Finally, a reverse pneumatic test was performed (with endoluminal serum insufflation, as pneumoperitoneum was established), and possible bleeding points were additionally assessed.

Protective ileostomy was performed in all patients in the series.

The technical steps are summarized in Figs. 1 and 2.

## Results

### Patient demographics

The intervention was performed on 22 patients—13 men (59%) and 9 women (41%)—with a mean body mass index of 29.6. Neoadjuvant therapy was administered to 13 of the 22 patients (those with locally advanced lesions at the time of diagnosis according to preoperative staging tests).

The demographic characteristics are summarized in Table 1.

**Table 1:** Demographic and preoperative characteristics

**Pa**	**Sex**	**Age**	**BMI**	**Comorbidities**	**Distance from anus (cm)**	**Neoadjuvant**	**Operative time** **(minutes)**
1	M	60	28	0	5	RT + FOLFOX	200
2	M	81	32	Hypertension	6		210
3	F	68	31	Hypertension	7		190
4	F	50	29	DM	7	RT + CAPEOX	220
5	F	71	38	DM, CKD	7	RT + FOLFOX	240
6	M	75	33	CKD	6	RT + FOLFOX	180
7	M	65	22	0	6	RT + FOLFOX	190
8	M	75	23	DM	8		200
9	M	81	19	Hypertension	5		210
10	M	57	36	Hypertension	5		220
11	F	72	37	0	6	RT + FOLFOX	320
12	F	47	40	DM	6	RT + FOLFOX	180
13	M	76	29	0	7		170
14	M	59	30	CKD	6	RT + FOLFOX	190
15	M	51	31	0	8	RT + FOLFOX	170
16	M	53	32	0	6		210
17	M	63	21	0	7	RT + FOLFOX	220
18	M	58	22	0	5		190
19	F	55	23	DM, hypertension	8	RT + FOLFOX	200
20	M	68	42	0	4	RT + FOLFOX	190
21	F	73	20	CKD	4		240
22	M	63	32,5	DM	6	RT + FOLFOX	190

*BMI* body mass index, *M* male, *F* female, *Hypertension *high blood pressure, *DM *diabetes mellitus, *CKD *chronic kidney disease, *RT *radiotherapy

### Tumour and operative characteristics

The mean distance to the tumour from the external margin was 6.1 cm, and a laparoscopic approach was used in all patients, and the mean surgery time was 206 min.

### Postoperative outcomes

 In all patients in the series, the mesorectum was completely removed, and there was no tumour involvement of the margins. The median postoperative length of stay was 5 days. Two patients in the series (9.1%) had AL (both with advanced local staging at the time of diagnosis and who received preoperative neoadjuvant therapy). These patients did not require reoperation and were instead treated with a rectal endosponge, with good subsequent evolution. In all patients, mesorectal excision was complete; the patients had tumour-free radial and distal margins, and none of these patients included in the series died. The rest of the posttrial results are summarized in Table 2.

**Table 2:** Postoperative results

**Pa**	**Postoperative stay**	**Complications**	**Clavien‒Dindo**	**Treatment**	**Re-entry (90 days PO)**	**Cause of readmission**
3	4				1	Dehydration
5	21	Anastomotic leak	IIIa	Endosponge		
9	5				1	Dehydration
15	4				1	Dehydration
20	18	Anastomotic leak	IIIa	Endosponge		

## Discussion

The treatment of rectal cancer continues to be a therapeutic challenge for multidisciplinary teams. In some patients after neoadjuvant treatment, a complete clinical response may be observed, with the disappearance of the tumour lesion, which has given way to the strategy of organ preservation without surgery, a strategy called ‘Watch and Wait’, with results in this group of patients apparently similar to those of surgery involving rectal resection and TME [12]. However, in patients experiencing persistent tumours after neoadjuvant therapy, surgery remains the cornerstone of treatment, with curative intent for rectal cancer. Healing and overall survival are the primary goals of surgery, but preserving the form and function of the sphincter is also important [13]. Surgery for tumours of the rectum is highly complex and technically demanding in many cases. Due to this complexity, multiple techniques and modifications have been developed in recent decades to improve oncological outcomes and reduce the incidence of complications, with AL being the most notable and causing the most morbidities [14].

In this quest to reduce the incidence of AL, single-stapled techniques for colorectal anastomosis after low anterior resection (TaTME was the first technique to be introduced) apparently considerably reduce the incidence of AL. An example of this was presented in a comparative study of patients treated with single-stapled (SS) techniques (TaTME and TTSS) versus the double-stapled technique (DS). The AL rate was 6.48% in the SS group (185 patients) and 15.28% in the DS group (*p* = 0.002).

Although the SS technique has a low AL rate, it is disadvantageous because the technical complexity of TaTME and the complications presented by some groups at the beginning of the technique were not common with the abdominal approach (e.g., prostatic urethral lesions) [15]; therefore, multiple groups have tried to maintain the demonstrated advantages of TaTME as well as simplify the formation curve and further reduce the complication risk.

As part of these efforts, in 2019, Prof. A. Spinelli published the first patient series, with 13 patients who underwent low anterior resection and TTSS and 7 with ileoanal reservoirs and who underwent TTSS; only four patients in the series experienced complications—one with Grade IIIa and the remaining three with Grade I (Clavien–Dindo classification) complications—with a minimum follow-up period of 6 months.

Subsequently, the same group published a report of a comparative series of 50 patients who underwent TTSS, 127 who underwent double-stapled resection and 100 who underwent TME. In this series, the AL rate was 17.5% in the DS cohort, 6% in the TME cohort, and 2% in the TTSS cohort (*p* = 0.005), emphasizing the potential of TTSS as a safe option for these patients.

This trend was recently confirmed by the results of the prospective IDEAL cohort study (stage 2 a/b) [16], in which of 275 patients with medium and lower rectal tumours were compared and divided into three groups: 70 patients who underwent surgery via the TTSS technique, 110 patients who underwent surgery via the DG technique, and 95 patients who underwent coloanal anastomosis. When comparing the data of patients who underwent TTSS with those of patients in the DS group, the AL rates were 8.6 and 20.9%, respectively (*p* = 0.028), with a similar incidence of low anterior resection syndrome (LARS) in both groups.

These results are comparable to those observed in our series since the introduction of the TTSS technique by our team. Of the 22 patients with tumours in the middle and lower rectum (mean distance to the anal margin of 6 cm in the series), AL occurred in only 2 of the patients (9.1% of the series, and the rate was very similar to that of the IDEAL study). Likewise, from an early oncological point of view, all patients in the series presented a validated TMS in the assessment of pathological anatomy and radial and distal margins free of tumour involvement.

Prof. Spinelli’s team [11] argues that TTSS has apparent advantages over TaTME, such as the fact that in the case of TTSS, the first purse-string is formed once the rectum and mesorectum have already separated from the pelvic floor, facilitating the preparation of the pouch and reducing the possibility of injuring nearby structures. In the case of rectotomy, the rectum is completely liberated, which facilitates sectioning, and the abdominal equipment can be used to help with transillumination. In the case of TTSS, the rectal cuff below the level of transection has already been completely dissected from the pelvic floor before sectioning. This allows the second-purse string to be formed without additional manoeuvres, which can increase the risk of complications and facilitate preparation of the anastomosis.

These advantages were also observed in our series, in which, with a shorter learning curve, an AL rate less than 10% was achieved (despite the early nature of the series), without the need for reoperation or permanent stomas and without patients treated with the TaTME approach presenting lesions or complications.

In conclusion, for all these reasons, the TTSS technique yields promising results and may be validated in subsequent studies; however, this technique should be considered a very valid option for patients with tumours of the middle and lower rectum.

### Limitations of the study

Despite the fact that these results were obtained early and from a series of centres, there are possible biases, such as the selection of cases or surgical teams.

## Data Availability

No datasets were generated or analysed during the current study.
